# Lanthanide Contraction in *Ln*F_3_ (*Ln* = Ce-Lu) and Its Chemical and Structural Consequences: Part 2: Specialized Empirical System of *R*^3+^ (*R* = Y, La, and 14 *Ln*) and F^1−^ Ionic Radii for *R*F_3_ Series

**DOI:** 10.3390/ijms242317080

**Published:** 2023-12-03

**Authors:** Boris P. Sobolev, Elena A. Sulyanova

**Affiliations:** Shubnikov Institute of Crystallography, Federal Scientific Research Centre “Crystallography and Photonics”, Russian Academy of Sciences, Leninskiy Prospekt 59, 119333 Moscow, Russia; sobolev.b@crys.ras.ru

**Keywords:** lanthanide contraction, ionic radii, empirical system of ionic radii, rare earth elements, lanthanide trifluorides, fluorine anion radius

## Abstract

A *specialized empirical* (***Spec-zd Emp***) *system of ionic radii* (**SIR**) for ***R*** = Y^3+^, La^3+^, *Ln*^3+^, and F^1−^ (*R* rare earth elements (**REE**)) was derived from the dependence of *lanthanide contraction* (**LC**) on the *atomic number* (**Z**) of *lanthanides* (***Ln***). LC decreased the radius of the cation with increasing Z. The structures of ***t***-*R*F_3_ (LaF_3_-NdF_3_, “*pseudo* ***t***-SmF_3_”) of the LaF_3_ type, 11 ***β***-*Ln*F_3_ (*Ln* = Sm-Lu), and ***β***-YF_3_ of the β-YF_3_ type were studied. The empirical basis of the shortest (F-F)_min_ and (*R*-F)_min_ distances was calculated from the structural data for the *R*F_3_ complete series. The dependence of (F-F)_min_ on Z reached saturation at Z = 67 (Ho). The base F^1−^ radius ***r***_−_ = 1.2539(16) Å was calculated as the arithmetic mean of five (F-F)_min_ in *Ln*F_3_ with *Ln* = Ho-Lu. For the *Ln*F_3_ series with *Ln* contributions up to 75 % wt., the dependence of (*Ln*-F)_min_ on Z reflected the non-uniformity of the 4*f* orbital filling. SIR was calculated as the difference in the empirical constants of *R*F_3_ (*ionic radii* of (*R*,*Ln*)^3+^ (***r***_+_) and F^1−^ (***r***_−_)), the change in which was continuous over the series and did not depend on the type of structure: ***r***_+_ = (_Z_*R*-F)_min_ − ½(F-F)_min_ (Z = 57–71). The changes in LC in the *Ln*F_3_ series were described by a third-degree polynomial. LC reduced ***r***_+_ by 24% (percentage relative to less) from 1.1671(16) Å (La^3+^) to 0.9439(17) Å (Lu^3+^). In the *Spec-zd Emp* SIR, ***r***_+_ were constants that did not require corrections for a *coordination number* (**CN**). A comparison of ***r***_+_ in the *Spec-zd Emp* SIR with other SIRs was performed.

## 1. Introduction

This work continues the study of LC and its effect on the structural and chemical properties of REE trifluorides *R*F_3_. In [1], the main chemical consequence of LC is the involvement of YF_3_, which is the fluoride of *d*-element, in a series of 14 fluorides of 4*f*-elements. LC generates structural and chemical affinities of members of the short (*d*-elements Sc, Y, and La) and long (La and 14 *f*-elements *Ln*) homologous series.

The series differ in terms of the electronic structure of the cations. Despite this fundamental difference, the chemical consequence of LC is the unification of part of a short with a long series into a new conglomerate of 16 *R*F_3_ (without ScF_3_) by structural and chemical proximity. The exclusion of ScF_3_ is due to its structure and properties being significantly different from other *R*F_3_ [2].

Despite the difference in the electronic structure, according to IUPAC recommendation, La is classified as *Ln*. This disadvantage of REE classification was noted by IUPAC as a project to clarify the location of La (2015). In this study, formula (La,*Ln*)F_3_ is used when discussing issues related to LC. This formula is not equivalent to a structural formula that isolates chemical elements in equivalent structural positions. The purpose of the (La,*Ln*) designation is to separate 14 *Ln* (4*f*-elements), which are exposed to LC, from La, which has no *f*-electrons. When LC is not discussed, the *R* abbreviations for Y, La, and 14 *Ln* are used.

The location of *d*-element fluoride _57_LaF_3_ before _58_CeF_3_ is determined by Z = 57 of La. The location of *d*-element fluoride _39_YF_3_ in the *Ln*F_3_ series is not set by Z = 39 of Y but by structural and chemical properties of YF_3_. The location of YF_3_ is different for the different properties, according to which the ion of *d*-element Y^3+^ is assigned the values of “*pseudo* Z_Y_” and ***r***_Y_. Based on the structural data of ***β***-YF_3_ [1], the location of YF_3_ between HoF_3_ and ErF_3_ with “*pseudo* Z_Y_” = 67.42 is determined.

To date, only the LaF_3_ and 10 *R*F_3_ structures (out of 16 without ScF_3_) have been studied. No data are available for *Ln*F_3_ with *Ln* = Pm, Gd, Dy, Tm, and Lu. Therefore, to clarify the location of YF_3_ according to its structural data in [1], it is necessary to study the structures of ***β***-*R*F_3_ crystals with *R* = Ho-Lu, Y at standard ***P***_st_ and ***T***_st_. All crystals are obtained under the same thermal conditions from the reagents purified from oxygen impurities. For the first time, the structures of all *R*F_3_s are investigated on crystals grown under the same thermal conditions in [1] and in this study.

Analysis of the *R*F_3_ structures studied to date has shown that they are unsatisfactory in terms of the number of compounds and reliability, particularly in early studies.

The study of *R*F_3_ structures has been ongoing for almost a century (since 1929). During this period, the crystal compositions have changed. During the 1930–1970s of the last century, the content of REE impurities in *R*F_3_ was high. In some cases, the main element accounted for only 50%. In 1971, *R*F_3_ (*R* = La, Pr, Nd, Gd, Ho, Lu) with a total REE content of 35–125 ppmw and other cations of 30–55 ppmw were obtained [3,4,5]. These *R*F_3_ structural data are unsuitable for calculation of a fully *specialized* (***Spec-zd***) *empirical* (***Emp***) *system of ionic radii* (**SIR**) (for *Ln*^3+^ cations and F^1−^ anion). We denote the proposed system as *Spec-zd Emp* SIR for *R*F_3_ (*R* = Y^3+^, La^3+^, *Ln*^3+^) and F^1−^.

The cationic impurity composition and uncontrolled factors, such as pyrohydrolysis, make some *R*F_3_ structures incomparable and the data on interatomic distances unreliable. Obtaining the structures of ***t***- and ***β***-*R*F_3_ (*R* = Y, La-Lu) for the precise study of LC evolution provides material for the next task of studying LC. Such a task is to create the *Spec-zd Emp* SIR for (La,*Ln*)F_3_ based on the change in ***r***_+_ in one *Ln*F_3_ series at LC. This SIR is the first to be completely (by anion and cations) empirically calculated. To calculate this, it is necessary to revise the structural data of all (La,*Ln*) F_3_ and YF_3_, which is one of the tasks in this series of studies.

The precise LC study requires structural data of the crystals of the entire *R*F_3_ series. The previously used inter- and extrapolations of missing *R*F_3_ structural data are unacceptable for such a study. To be comparable, the data must be obtained from crystals grown at a modern technological level (with an absence of isomorphic oxygen). Structural analysis should be performed using modern equipment and programs to process the results.

In this study, five ***t***-*R*F_3_ (LaF_3_-NdF_3_, “*pseudo* ***t***-SmF_3_” of the LaF_3_ type [6,7,8]), 11 ***β***-*Ln*F_3_ (*Ln* = Sm-Lu), and ***β***-YF_3_ of the ***β***-YF_3_ type [9]) are studied. The structural data of “*pseudo* ***β***-PmF_3_” and “*pseudo* ***t***-PmF_3_” are taken from the previous work [10].

Together with the structural data for ***β***-*R*F_3_ (*R* = Ho-Lu, Y) [1], precise structural data of an almost complete series of *R*F_3_ crystals are obtained. They are suitable for calculating the empirical values of the ***r***_−_ radius from the dependencies on Z of the shortest (F-F)_min_ distances and ***r***_+_ radii from the shortest (*R*-F)_min_ in the (La,*Ln*)F_3_ and YF_3_ structures. On their basis, the dependence of LC on Z is constructed, reflecting the interaction in the “*Ln* nucleus-4*f*-electrons” subsystem, and *Spec-zd Emp* SIR for (Y,La,*Ln*)F_3_ is calculated.

This study aims to investigate the structures of *R*F_3_ with *R* = La-Nd, ***t***-Sm_0.995_La_0.005_F_3_ (“*pseudo* ***t***-SmF_3_”), and ***β***-*R*F_3_ with *R* = Sm-Dy by using them and the structural data of *β*-*R*F_3_ (*R* = Ho–Lu, Y) [1], “*pseudo* ***t***-PmF_3_”, and “*pseudo* ***β***-PmF_3_” [10] to construct completely empirical *Spec-zd Emp* SIR for Y^3+^, La^3+^, and 14 *Ln*^3+^ and F^1−^ for *R*F_3_.

## 2. Results and Discussion

### 2.1. Lanthanide Contraction of Ln^0^ Elements and Ln^3+^ Ions

**Figure 1:**
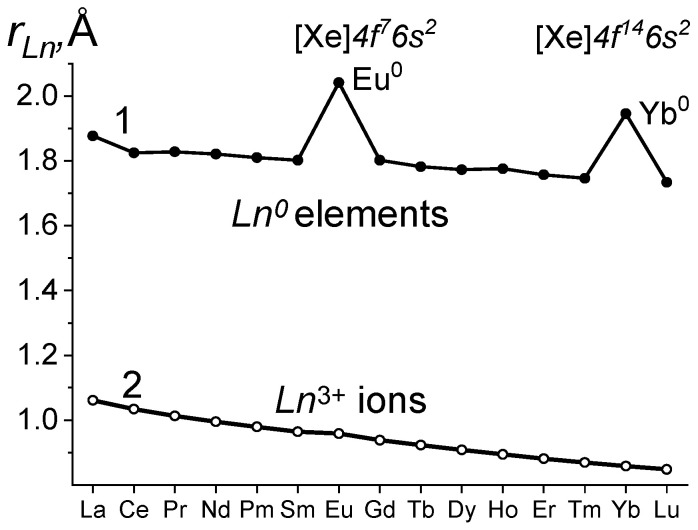
Lanthanide contraction: curve 1, *Ln*^0^ elements; curve 2, *Ln*^3+^ ions (reference data).

LC was first discovered in 1926 [11]. It has been studied for almost a hundred years. For elements (*Ln*^0^), LC is described as a monotonic decrease in the radii with deviations in Eu^0^ and Yb^0^ (curve 1 in Figure 1 (reference data)). They are associated with stable configurations of the electronic shells in Eu^0^ *4f*^7^*5d*^0^*6s*^2^ и Yb^0^ *4f*^14^*5d*^0^*6s*^2^.

For *Ln* compounds, changes in ***r***_+_ with LC are not described so unambiguously. Curve 2 in Figure 1 is one of the options for ***r***_+_ dependence on Z, according to the reference data (the chemical class of compounds is not specified). It has a bend at Eu^3+^. In the series of other compounds, the bend may be displaced or absent. The “outlier” of Gd or its possible “contribution” to the radii of subsequent *Ln*^3+^ ions has most often been discussed in the literature.

The *Ln*^3+^ CN is not discussed. The CN is considered constant for all *Ln* compounds of the same type of structure. However, this is not the case for ***β***-*Ln*F_3_ crystals. In the β-YF_3_ (anti-Fe_3_C) type, CN is variable [12] and can take non-integer values.

The CN corrections for *Ln*^3+^ in the *universal* (***Univ***) SIR [13,14] have not been defined. To reasonably introduce this correction, it is necessary to study the structure beforehand and determine the real CN of the cation in the selected *Ln*F_3_.

Curve 2 in Figure 1 does not reflect the variety of properties of the homologous series of *Ln* compounds of different chemical classes that are dependent on Z. Most of the dependencies of ***r***_+_ on Z lead to the conclusion that, in *Ln* compounds, LC in its volumetric expression is close to the monotonic dependence on Z. This contradicts the known and observed changes in LC and properties of *Ln* compounds belonging to a single homologous series.

**Figure 2:**
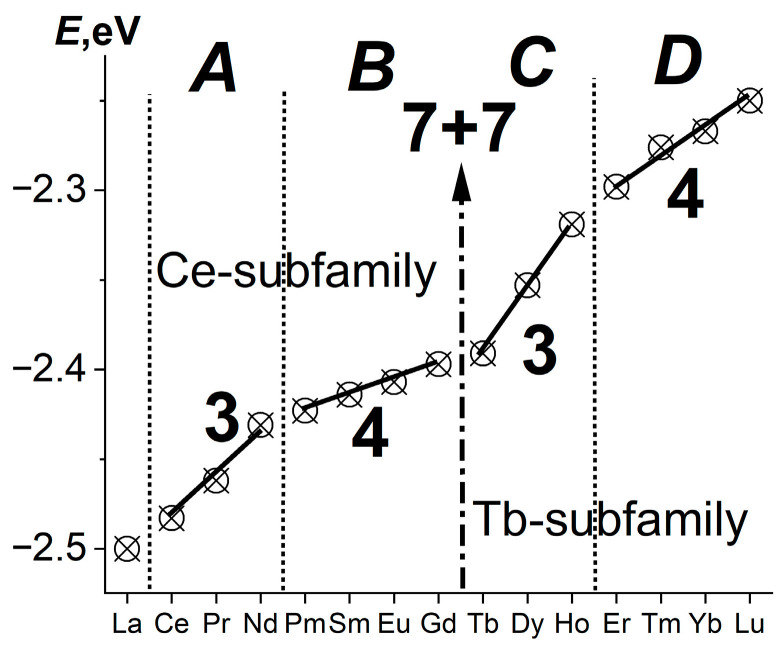
Standard electrode reduction potentials of La and *Ln* in a solution (reference data). The structural subgroups of (La,*Ln*)F_3_ are denoted by the letters ***A***–***D***. The number of *R*F_3_ in each structural subgroup (3 in ***A*** and ***C*** and 4 in ***B*** and ***D***) is shown near the curve.

Figure 2 shows the changes in the number of *Ln* standard electrode reduction potentials in the solution (reference data). These changes reflect the 7 + 7 internal periodicity of the *Ln* series (the division into Ce and Tb subfamilies of seven elements (a consequence of Hund’s rule)).

The intraperiodic periodicity of the phase transformations superimposed on the internal periodicity is also clearly reflected in Figure 2 [15]. It divides each subfamily into two parts containing 3 and 4 *Ln*. The reason for this has not yet been established. In Figure 2, it is reflected by the letters ***A***–***D***, denoting four structural subgroups of (La,*Ln*)F_3_ resulting from division.

A similar division of the *Ln* series into four parts reveals a more fundamental property: the ionization potentials of atoms Ln^0^ → *Ln*^3+^ + 3e^−^. However, the authors [16] describe the dependence they obtain, similar to Figure 2, as “uniformly increasing”.

The uniform and close to linear dependence of ***r***_+_ on Z (Figure 1) may mean that the specifics of filling the 4*f* orbital are not reflected in LC. This is contradicted by LC studies on the properties of one homologous series of compounds. Reduction and ionization potentials are not the only properties of *Ln* compounds with such dependencies on Z.

The uniformity of *Ln*^3+^ LC in different homologous series that are not united in an array has been little studied. Therefore, widespread ideas about LC do not reflect real situations.

### 2.2. LC in Large Arrays of Ln Compounds and the Variability of the Ln^3+^ Radii

Large arrays of *Ln* compounds with different chemical bonds mask the effect of the 4*f* orbital on the changes in ***r***_+_ in the *Ln*F_3_ series caused by LC. Averaging over the series makes the dependence “pseudo-monotonic”. Based on this dependence, it is impossible to obtain the precise data necessary for the study of LC.

Because obtaining the correct data for constructing the *Sp-zd Emp* SIR for (La,*Ln*)F_3_ depends on the solution of the uniformity problem of the ***r***_+_ dependence on Z, we will pay some attention to it.

The full homologous series of 17 REE compounds is the longest series of inorganic compounds. After solving the problem of REE separation, a rapid increase in the number of new *Ln* compounds is observed. There is a tradition of studying (or comparing) the LC of several series of compounds at the same time. It has been believed that the analysis of arrays of series of compounds gives a statistically more reasonable evolution of LC in the series of *Ln* compounds. This should be the case if the measured ion characteristics (for example *Ln*^3+^ radii) are constant and comparable.

The arrays of compounds provide distorted information. The mechanism of LC is concentrated in the interactions in the intra-atomic subsystem “*Ln* nucleus—4*f* electrons”. The subsystem is shielded but not isolated from the interaction with valence electrons, contributing to the chemical bond. Differences in the magnitude of the contribution to the chemical bonds of *Ln* with different anions lead, under the action of LC, to “blurring” of the boundaries of the change in structural types in different chemical classes of *Ln* compounds.

The most fundamental monograph dedicated to LC studies describes the structures of 400 isostoichiometric series of *Ln* compounds obtained by 1984 as complex, oxygen, intermetallic, chalcogenide, and halide (in decreasing order of number) [17]. The totality of many series of REE compounds as complex and “unknown” chemical systems is considered in [17]. This definition is concluded [17] as a result of the analysis of the unique volume of structural material on LC.

Four regions with “blurred” boundaries are identified [17]. They are called areas of crystal chemical instability. Changes in the type of structure are most likely to occur in them. The distribution of the three positions of change in the type of structure across the series of compounds is statistical in nature: Nd-Pm (20.6%), Gd (10.7%), and Dy-Ho (18.4%) out of 364 cases. In a particular series, there may be two, one, or no changes in the structure.

In [17], a number of important conclusions regarding the topic of LC are made. Since the book has not been translated into English and is therefore little known, we will briefly outline the conclusions made by the authors. They are directly related to the manifestation of LC in the *Ln*F_3_ homologous series *Ln* compounds.

The smooth ***r***_+_ change expected in the *Univ* SIRs [17] is not observed. The authors conduct a detailed analysis of all the structures of the *Ln* compounds studied by 1984. The average values of ***r***_+_ for CN from 6 to 12 are calculated. The *Ln*-O distances in the polyhedra with the same CN are averaged. The shapes of polyhedra do not affect the average *Ln*-O distances.

Based on the unique volume of collected and analyzed structural data [17,18,19,20], a conclusion is made about the relativity of the concept of “ionic radius” for REE. The radii of individual *R*(*Ln*)^3+^ ions lose their permanence when used to calculate the average radii of REE ions from a series of REE compounds with different structures. This contradicts the prevailing opinion in the literature about the uniform dependence of LC on Z. The conclusion is very fundamental; the reason for the uneven character of ***r***_+_ dependency on Z is not the low accuracy of structural definitions but the difference in the interatomic distances.

The authors [17] conclude the section by obtaining REE ionic radii on the base of analysis of arrays of homologous series of REE compounds of different chemical classes with the phrase: “It is impossible to obtain an ideal system of ionic radii of REE by averaging interatomic distances taken from a large number of structural definitions”. This is equivalent to the statement that the ionic radii of *R*(*Ln*)^3+^ are not constants for an individual element, rather they reflect different structural and chemical states in crystals with different chemical bonds.

The authors [17] believe that it makes no sense to calculate SIR for *Ln*^3+^ with an accuracy of ±0.001 Å. Below are the reasons to believe that this conclusion [17], which has received exhaustive statistical confirmation, is incorrect because of the chemical diversity of large datasets. Usually, *Ln* compounds are selected in a large array for analysis, not by the nature of the chemical bond but by the degree of study of the series. The most fully studied series are preferred, since only in them is there hope to notice deviations from the homogeneous change in LC.

The selection of structures for analysis in [17] is based on this principle. With incomplete series, missing data are obtained by interpolation (extrapolation) for a number of compounds with the studied properties, which is unacceptable for the precise study of LC. Therefore, in [1] and in the present work, it is necessary to study the structures of 18 *R*(*Ln*)F_3_ crystals of the same series, with chemical affinity obtained under the same thermal conditions.

When analyzing large arrays of *Ln* compounds that are inhomogeneous in chemical bonding, the contributions of the non-monotonic filling of the 4*f* orbital, its interactions with valence electrons (contribution to the chemical bond), different packing densities of different types of structures, and other factors remain unexplored or undivided.

The specificity of the (La,*Ln*)F_3_ series (ΔZ = 1 for all neighboring La and *Ln* elements with Z ranging from 57 to 71) provides a high relative accuracy for (La,*Ln*)^3+^ radii. This accuracy is unattainable in any *Univ* SIR that uses large arrays of chemically dissimilar *Ln* compounds.

The spin–orbit coupling effect in the 4*f*- orbital of *Ln* might play a role in the LC non-uniformity. The discussion of the 4*f* orbital spinor structure as a cause of LC non-uniformity is beyond the scope of this study.

The difference in the types of chemical bonds in different homologous series “blurs” the dependence of LC on Z. The nature of a bond can vary significantly within a single compound. This is especially typical for heterodesmic complexes, the number of which is very large in *Ln*.

In another fundamental study of the problem of ionic radii [21], the authors come to a similar negative conclusion: “The limiting values of ionic radii cannot be directly used to calculate interatomic distances in crystals with an intermediate bond character”. This is equivalent to the following statement: for compounds with an intermediate nature of chemical bonding, the ionic radii cannot be calculated from interatomic distances. “They (ionic radii) may be useful for theoretical assessments of the ionic state of substances” [21]. The degree of chemical bond ionicity required for calculating the radii is not specified in [21].

It follows from [21] that substances with ionic bonds are not rigidly subject to this restriction. Moreover, high ionicity of chemical bonds can provide the characteristic of a substance. Perhaps one of these characteristics is the shortest (*R*-F)_min_ distance in ionic *R*F_3_, which is individual for each *R*^3+^. The contribution of ***r***_−_ to them is constant for a series, and the contribution of cations is individual and corresponds to their “spheres of influence” in a “cation-anion” pair, as in the case of individual “spheres of influence” in an “anion-anion” pair [22].

The corrections for the degree of chemical bond ionicity described in all the *Univ* SIR are performed according to Pauling’s electronegativity scale (1932). Its value for fluorine, according to the modern system of thermochemical electronegativities [23] (4.0), coincides with the value of 3.98 defined by Pauling. At both scales, REE fluorides have some of the most ionic bonds.

The categorical nature of the remarks [21] seems to be caused by the fact that they are made in relation to the *Univ* SIR, which uses compounds with different chemical bonds. Large arrays of homologous series of *Ln* compounds with different types of chemical bonds are unsuitable for precise analysis of LC and obtaining the *Spes-zd Emp* SIR for Y^3+^, La^3+^, and *Ln*F_3_.

The conclusion follows from the above: the evolution of LC must be studied within the framework of a homologous series of *Ln* compounds of one chemical class with a high degree of ionicity of their chemical bonds. The series of 17 *R*F_3_, with its constituent part of 15 (La,*Ln*)F_3_, is one of the longest homologous series of inorganic compounds.

Important conclusions based on large statistics [17,21] lead us to abandon the average values of the interatomic distances. The shortest (*R*-F)_min_ and (F-F)_min_ distances are used to create the *Sres-zd Emp* SIR for Y^3+^, La^3+^, 14 *Ln*^3+^, and F^1−^ in (*R*,*Ln*)F_3_.

### 2.3. Advantages of the (La,Ln)F_3_ Series for Precise Study of LC and Obtaining r_+_ and r_−_

The series that includes LaF_3_ and 14 *Ln*F_3_ are the most suitable for creating the *Spec-zd Emp* SIR for Y^3+^, La^3+^, 14 *Ln*^3+^, and F^1−^ ions among numerous homologous series of inorganic *Ln* compounds. This series is distinguished by features that make it unique for the precise study of LC. The general formula for the *R*F_3_ series is extremely simple. It contains qualitative and quantitative compositions that are favorable for the precise study of the LC evolution.

The F^1−^ anion has one of the lowest atomic masses (19) among simple anions. High atomic masses of *Ln*^0^ lead to the fact that, at the beginning of the series, CeF_3_ has more than 71%, and, at the end of the series, LuF_3_ has ~75% of the *Ln*F_3_ mass accounted for compressible *Ln*^3+^ cations and only 25–30% for anionic “ballast”. In all *Ln* salts and oxygen-containing acids, the mass contributions of *Ln* are lower. For comparison, the wt.% of Lu in the *Ln*WO_4_Br series [24] is ~35%. As a result, the *Ln*F_3_ series has an abnormally high “sensitivity” of LC to the peculiarities of filling the 4*f* orbital.

The consequence of the high chemical bond ionicity is the high melting points (1143–1552 ± 10 °C) and chemical stability of *R*F_3_. All *R*F_3_ crystallize from the melt. Eight of them are obtained in the form of single crystals (ScF_3_, LaF_3_-NdF_3_, TbF_3_-HoF_3_), and another five (GdF_3_, ErF_3_-LuF_3_) in the form of large blocks suitable for structural studies.

Polymorphic transformations are inhibited in SmF_3_ and EuF_3_. The grain sizes of the modifications strongly depend on the cooling conditions and presence of impurities. Because of the lack of high-quality crystals for structural analysis, ***t***-EuF_3_ and ***t***-GdF_3_ have not been characterized in the present study.

PmF_3_, which is inaccessible for research, is obtained by structural and chemical modeling [10]. It is named “*pseudo* _61_PmF_3_” because it differs from PmF_3_ by composition ^61^(^58^Ce_0.5_^64^Gd_0.5_)F_3_, but it has the same average Z = 61 as _61_Pm. In terms of the structural and thermal properties, the model duplicates inaccessible PmF_3_. We also use structural and chemical modeling for the non-quenchable modification of ***t***-SmF_3_.

### 2.4. Lande’s Empirical Approach to Determining the Radius of an Anion

SIRs began to be created in the 1920s of the last century, with research by Lande [22]. Because the radius of no ion was known, Lande proposed to use empirical values (unit cell parameters) to determine the sizes of large anions.

According to [22], ions are incompressible spheres, between which only Coulomb forces act. When the distance between the ions is equal to the sum of their radii, infinitely large repulsive forces arise. The limit of anions convergence is called the “sphere of influence”. The radius of an anion is equal to its half. The radii of the anions obtained by Lande’s method are used to calculate the radii of the cations in many *Univ* SIRs.

One homologous series is a prerequisite for obtaining the precise characteristics of the LC and data for the *Spec-zd Emp* SIR. This condition is called [1] the internal matching of cations and the anion by the source of obtaining characteristics (one (La,*Ln*)F_3_ series). The use of a single homologous series eliminates the “blurring” of LC that occurs in arrays of different homologous series of *Ln* compounds because of the difference in the nature of their chemical bonds [17].

The source of information about the radii of the cations in *R*F_3_ is a group of empirical quantities (interatomic distances (*R*-F)_min_). In the present study, precise X-ray diffraction data for 18 *R*F_3_ samples are obtained. Two modifications (***t***- and ***β***-) are studied for PmF_3_ [10] and SmF_3_. The structures of six trifluorides, from HoF_3_ to LuF_3_ and YF_3_, are given in [1]. This study presents structural data for ***t***-*R*F_3_ (*R* = La-Nd), “*pseudo* ***t***-SmF_3_”, and ***β***-*Ln*F_3_ (*Ln* = Sm-Dy).

### 2.5. Determination of r_−_ from the (F-F)_min_ Dependency on Z in the (La,Ln)F_3_ Series

The first task of this work is to find (F–F)_min_ that satisfy the condition of Lande’s “spheres of influence”. We will follow this approach: the search for the shortest distance among the shortest (F-F)_min_ in the (La,*Ln*)F_3_ series.

Repeating Lande’s approach [22] to calculate ***r***_−_ for *Spec-zd Emp* SIR from (F-F)_min_ requires two conditions: (1) the use of one homologous series and (2) the presence of a minimum on the (F-F)_min_ dependence on Z or its saturation. (F-F)_min_ is the sum of ***r***_F_: (F-F)_min_ = 2·***r***_−_.

The (F-F)_min_ and (*R*-F)_min_ obtained from the structural studies in the present work and in [1,10] are listed in Table 1.

**Figure 3:**
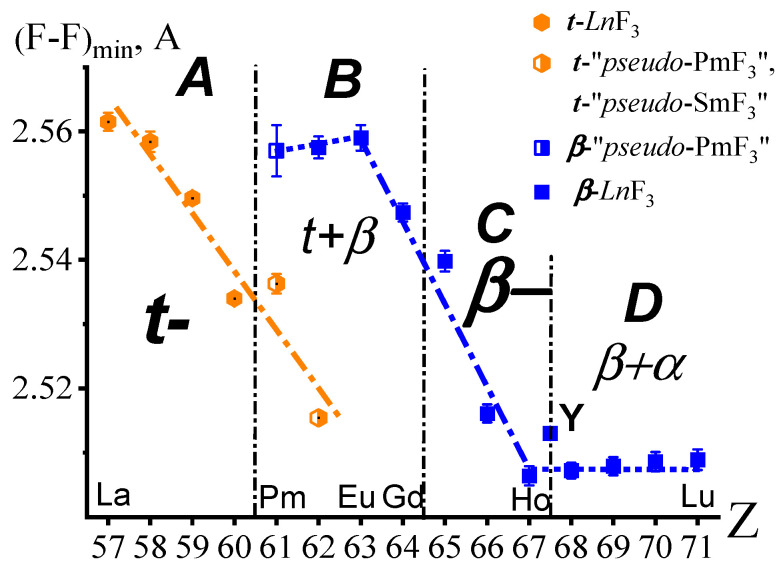
(F-F)_min_ in ***t***-*R*F_3_ (*R* = La-Nd), “*pseudo* ***t***-PmF_3_”, “*pseudo* ***t***-SmF_3_”, “*pseudo* ***β***-PmF_3_”, ***β***-YF_3_, and ***β***-*Ln*F_3_ (*Ln* = Sm-Lu). The structural subgroups of (La,*Ln*)F_3_ are denoted by the letters ***A***–***D***.

The dependence of (F-F)_min_ on Z in the ***t***-(*R*,*Ln*)F_3_ structures is shown in Figure 3 by orange closed hexagons for *R* = La-Nd and semi-open hexagons for “*pseudo* ***t***-PmF_3_” and “*pseudo* ***t***-SmF_3_”. This dependence is linear and demonstrates a decrease in (F-F)_min_, with an increase in Z from _57_La to _62_Sm. The dependence of (F-F)_min_ on Z for ***β***-*Ln*F_3_ (*Ln* = Sm-Lu) is shown by blue closed squares and for “*pseudo* ***β***-PmF_3_” by a half-opened blue square.

In ***β***-modifications of dimorphic *Ln*F_3_, (F-F)_min_ exceeds similar distances in ***t***-modifications by ~1.2%. This is one of the manifestations of negative thermal expansion at polymorphic transformation [25,26,27].

In both types of structures, (F-F)_min_ decreases with the growth of Z along inclined (almost parallel) straight lines. For the ***β***-type, the dependence reaches saturation at Z = 67 (Ho). The absence of the structures of the ***t***-EuF_3_ and ***t***-GdF_3_ with inhibited polymorphic transformation does not allow us to conclude that there is a similar plateau for the ***t***-type structure.

The “sphere of influence” of fluorine anions for ***β***-*Ln*F_3_ (*Ln* = Sm-Lu) is reached at Z = 67 (Ho). The radius ***r***_−_ = 1.2539(16) Å is calculated from (F-F)_min_ on Z dependence as half of the arithmetic mean of the five experimental (F-F)_min_ values for *Ln*F_3_ (*Ln* = Ho-Lu).

### 2.6. The Shortest (R-F)_min_ Distances in (La,Ln)F_3_

**Figure 4:**
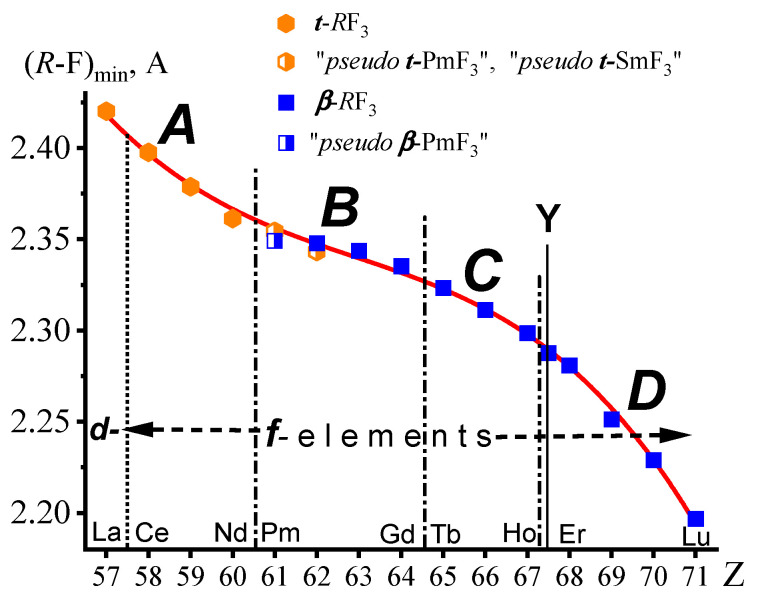
(*R*-F)_min_ in ***t***-*R*F_3_ (*R* = La-Nd), “*pseudo* ***t***-PmF_3_”, “*pseudo* ***t***-SmF_3_”, “*pseudo* ***β***-PmF_3_”, ***β***-*Ln*F_3_ (*Ln* = Sm-Lu), and ***β***-YF_3_. The structural subgroups of (La,*Ln*)F_3_ are denoted by the letters ***A***–***D***.

The dependence of (*R*-F)_min_ on Z for (La,*Ln*)F_3_ is shown in Figure 4. All the experimental points are fitted with a continuous function, which is a third-degree polynomial (1):

(*R*-F)_min_ = −1.67322·10^−4^·Z3 + 0.03163·Z2 − 1.99992·Z + 44.6499 (1)

The types of structures from which (*R*-F)_min_ are defined do not affect the dependence of (*R*-F)_min_ on Z. The (*R*-F)_min_ distances for the two forms of PmF_3_ and SmF_3_ (Figure 4) differ within the error. Equal (*R*-F)_min_ in PmF_3_ and SmF_3_ ***t***- and ***β***-modifications are a consequence of the manifestation of LC in the *Ln*F_3_ series. At the same time, the minimum “cation-anion” distance varies in both modifications continuously (within the error).

The dependence of (_Z_*R*-F)_min_ on Z precisely reflects the evolution of LC in the (La,*Ln*)F_3_ homologous series. The accuracy is determined by standard deviation for (*R*-F)_min_, which does not exceed ±0.0017 Å in the studied structures.

### 2.7. Calculation of the Sres-zd Emp SIR of R^3+^ (R = Y, La, and 14 Ln) and F^1−^ for (R,Ln)F_3_

The *Spec-zd Emp* SIR is limited to 16 cations: Y^3+^, La^3+^, 14 *Ln*^3+^, and one F^1−^ anion. The number of the cations is determined by the periodic table of elements. Fourteen *Ln* are allocated in its sixth period according to their electronic structure, occupying one cell together with _57_La. Paired combinations of neighboring *Ln* in the series are separated by ΔZ = 1. This is a condition of the maximum chemical proximity of *Ln* and their compounds.

In this study, the *Spec-zd Emp* SIR is created for the first time for *R*^3+^ cations and F^1−^ anion. It is proposed to call this completely empirical, in contrast to the *Univ* SIRs. It is based on the same empirical structural parameters used by the *Univ* SIR (interatomic distances determined by structural analysis).

The accuracy of any empirical SIR is limited by the accuracy of structural analysis. The latter, as shown here for 18 *R*F_3_, is about ±10^−3^ Å. It is lowered in the *Univ* SIRs by corrections to radii for greater universality. The differences between the *Spec-zd Emp* SIR and *Univ* SIRs are in the selection of the types of interatomic distances, their arrangement, and processing.

In the 18 *R*F_3_ structures investigated in this study, (*R*-*R*)_min_ far exceeds the possible sum of the radii of the cations. For *R*^3+^ in *R*F_3_, it is impossible to determine ***r***_+_ from (*R*-*R*)_min_ using the “spheres of influence” of cations because their contact does not occur.

The second type of the shortest interatomic distances, which include the radii of the cations, is (*R*-F)_min_. This yields empirical ***r***_+_ values. To date, these distances have not been used to create completely empirical (by cations and the anion) SIRs.

It is shown in the *Sres-zd Emp* SIR for the first time that (*R*-F)_min_ is an individual characteristic of *R*F_3_. Together with the (F-F)_min_ distances, (*R*-F)_min_ forms the main distinguishing feature of the *Spes-zd Emp* SIR (the internal consistency of the radii of cations and the anion). This means that the base radii ***r***_−_ and ***r***_+_ are determined from a single row of compounds *R*F_3_, which is a common source of ***r***_+_ and ***r***_−_. Internal consistency distinguishes the *Spes-zd Emp* SIR from all the *Univ* SIRs, which are only partially empirical.

The value of ***r***_+_ obtained from the empirical (*R*-F)_min_ distances is not arbitrary. This is confirmed by the independent definitions of ***r***_−_ in *R*F_3_ by the Lande method [22]. The independent ***r***_−_ value for the *Spec-zd Emp* SIR is derived from the empirical (F-F)_min_ parameters of the *R*F_3_ structures. The ***r***_+_ + ***r***_−_ sum is additive if ***r***_−_ is the same for all *R*F_3_. If such ***r***_−_ is found and the additivity of (*R*-F)_min_ is observed, the full *Spec-zd Emp* SIR for *R*^3+^ and F^1−^ can be calculated as the difference in the empirical constants of *R*F_3_ crystal structures obtained under the same conditions:

***r***_+_ = (_Z_*R*-F)_min_ − ***r***_−_ (2)

The distance to which the cation allows the anion to approach itself (with a constant “sphere of influence” for all *R*F_3_) varies continuously along the *R*F_3_ series.

The cation radii ***r***_+_ calculated from the shortest distances (*R*-F)_min_ for each *R*F_3_ are given in Table 2. The standard deviations for ***r***_+_ in *R*F_3_ do not exceed ±0.0017 Å. The standard deviations for ***r***_+_ in the “*pseudo* ***β***-PmF_3_” and “*pseudo* ***t***-SmF_3_” model crystals do not exceed ±0.004 and ±0.0018 Å, respectively.

**Figure 5:**
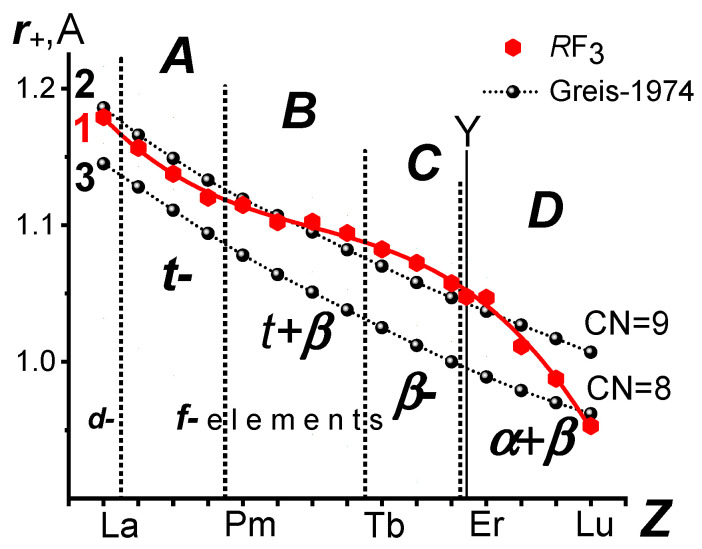
The *Spes-zd Emp* system of Y^3+^, La^3+^, and *Ln*^3+^ ionic radii ***r***_+_ with ***r***_−_ = 1.2539(16) Å. Curve 1 is the data of this study, curves 2 and 3 are the data of the Greis SIR [28] for (La,*Ln*)^3+^ with CN 9 and 8, respectively. The structural subgroups of (La,*Ln*)F_3_ are denoted by the letters ***A***–***D***.

The dependence of ***r***_+_ on Z according to the data obtained in this study is shown in Figure 5 by red closed hexagons. Curve 1 in Figure 5, which describes this dependence, is shown in red. Its shape is similar to the shape of the curve, which describes the change in (*R*-F)_min_ from Z in Figure 4, as these curves differ by the constant ***r***_−_. The dotted verticals indicate the boundaries between *d*-LaF_3_ and 14 *f*-*Ln*F_3_ and structural subgroups ***A***–***D*** having different types of structures (***t***-, ***β***-, ***α***-).

The non-uniformity of the ***r***_+_ dependence on Z (curve 1 in Figure 5) correlates well with the position of the boundaries between the structural subgroups (***A***–***B***) and (***C***–***D***).

The nonlinear dependence of ***r***_+_ on Z reflects the nature of the LC dependence on Z in the series of *Ln* compounds. In this study, for the first time, the relationship between LC and the volumetric effects of filling the 4*f* orbital is revealed using the empirical structural data of a single homologous series of (La,*Ln*)F_3_ ionic fluorides.

Comparison of the total effect of LC in the range from La^3+^ to Lu^3+^, according to different data, reveals a large difference. According to V.M. Goldschmidt, LC reduces ***r***_+_ for a number of REE oxides by 15% (percentage relative to less). According to the *Univ* SIR [14], for CN = 8, ***r***_+_ decreases by 16%. According to the *Spes-zd Emp* SIR, LC reduces the ***r***_+_ in (*R*,*Ln*)F_3_ by 24% (percentage relative to less) from 1.1671(16) (La^3+^) to 0.9439(17) Å in Lu^3+^.

### 2.8. Comparison of r_+_ in the Spec-zd Emp SIR with r_+_ in SIR [28]

The most cited *Univ* SIR [14] in part of REEs’ cations is based on the *r*_+_ radii proposed by [28]. To make these *R*^3+^ radii universal, amendments are made to them in [14]. They are different for cerium (+0.030 Å) and yttrium (+0.025 Å) subgroups of elements for CN = 9. For CN = 8 for all *R*^3+^, the correction is +0.015 Å. The amendments produce a “hand-made” inflection of the ***r***_+_ dependence on Z for CN = 9 in the PmF_3_ region.

Let us compare our *Spec-zd Emp* SIR to the original ***r***_+_ values from the SIR [28]. In Figure 5, the dependences of ***r***_+_ on Z in SIR [28] for CN 9 (curve 2, black balls) and CN 8 (curve 3, black balls) are shown. Curves 2 and 3 [28] in Figure 5 describe the change in ***r***_+_ from Z in the ***t***-(CN 9 according to [28]) and ***β***-(CN 8 according to [28]) structural types, respectively.

The ***r***_+_ values of curve 1 for *R* = La-Tm lie near the corresponding values of curve 2. Curve 1 intersects the dependence for SIR [28] for CN 9 (curve 2) twice. The first intersection point lies between Z = 62 (Sm) and 63 (Eu), the second lies between Z = 68 (Er) and 69 (Tm).

For *R* = La-Pm, ***r***_+_ lies below, and for *R* = Eu-Er lies above curve 2. In the Z region, from 69 (Tm) to 71 (Lu), the difference between the ***r***_+_ of the two SIR increases. Curve 1 approaches curve 3 for SIR [28] for CN 8 and intersects it between Z = 70 (Yb) and 71 (Lu).

The deviation of the ***r***_+_ dependence on Z in the *Spec-zd Emp* SIR from the linear law is a fundamental difference from the SIR [28] for *R*^3+^. The nonmonotonic nature of the ***r***_+_ dependence on Z is associated with the variable CN of *R*^3+^ in the *R*F_3_ series [12] and the presence of a minimum on the dependence on Z of the rhombohedral unit cell parameter *c* of ***β***-*Ln*F_3_.

This non-uniformity most likely reflects the volumetric changes in the construction of the 4*f*-orbital associated with its spinor structure.

### 2.9. Application of the Spec-zd Emp SIR for RF_3_ and RF_3_-R’F_3_ Systems

The complete (for *R*^3+^ and F^1−^) *Spes-zd Emp* SIR of Y^3+^, La^3+^, and *Ln*^3+^, and F^1−^ ionic radii ***r***_+_ and ***r***_−_, is intended for calculations in the fields of chemistry and crystal chemistry of Y, La, and 14 *Ln* fluorides. Their number is limited from 17 to 16 (without Sc). REEs number approximately 20% of the metal elements of the periodic table. For this number of elements and their compounds, the new SIR has no replacements.

There are many fluoride *R*F_3_-*M*F_n_ (*M* = Li, Na, K, Rb, Ca, Sr, Ba, Cd, Pb, Zr, Hf, Th; n = 1 ÷ 4) systems and materials based on *R*F_3_ [29]. If the second components are also limited to *R*F_3_, an array of 136 *R*F_3_-*R*’F_3_ systems is formed from 17 REE trifluorides, including “*pseudo* PmF_3_”. This is the main application of the *Spes-zd Emp* SIR for *R*F_3_.

For many years, the structural classification of *R*F_3_ and the associated chemical classification of *R*F_3_-*R*’F_3_ systems have been absent. This is due to the lack of polymorphism data for PmF_3_, which is unavailable for experiments. Thanks to the proof of the “*pseudo* _61_PmF_3_” polymorphism and its position in the SSGr ***B*** [10], a number of studies of *R*F_3_ and the *R*F_3_-*R*’F_3_ systems formed by them have become possible.

In the *R*F_3_-*R*’F_3_ systems, isomorphic substitutions are the only type of high-temperature interactions. In the studied systems, no formation of chemical compounds with structures different from the components are observed. However, the change in the chemical proximity of *R*F_3_ in the homologous series is quite large. This is reflected in the areas of homogeneity of the solid solutions and in the dependence on ΔZ of the cations of the systems components in which these solid solutions are formed.

The 34 systems studied are sufficient to calculate the singular points of many unexplored phase diagrams using the *Spes-zd Emp* SIR for (*R*,*Ln*)F_3_, which are the mutual solubility limits, compositions, and temperatures of phase reactions and morphotropic transformations.

With the high relative accuracy of ***r***_+_ in the *Sres-zd Emp* SIR, the errors of the theoretical calculations are comparable to the accuracy of differential thermal analysis in the study of phase diagrams. This makes it fast and efficient to fully characterize the high-temperature chemical interactions in *R*F_3_-*R*’F_3_ systems within certain limits of ΔZ. Restrictions on such calculations are imposed by large values of ΔZ of cations in the *R*F_3_-*R*’F_3_ systems (from 8 to 14).

Multicomponent fluoride crystalline REE-containing materials have been widely used in photonics. There is a tendency to increase the number of components and the use of three-component (*R*,*Ln*)F_3_-(*R*,*Ln*)’F_3_-(*R*,*Ln*)”F_3_ systems for matrix compositions. Multiple activations by REE ions (for example, for up-converters) significantly increase the number of possible fluoride materials. The *Spec-zd Emp* SIR of ***r***_+_ and ***r***_−_ is required for the structural and chemical analysis of REE-containing crystals and the prediction of the phase diagrams of the chemical systems formed by them.

### 2.10. Prospects for the Expansion of the Spes-zd Emp SIR for (R,Ln)F_3_ to Ionic Inorganic Fluorides

Phase diagrams represent the fundamental physicochemical basis of the chemistry of inorganic fluoride binary systems. These systems are the simplest of multicomponent ones. Knowledge of their phase diagrams allows us to predict the phase composition of the next stage of complexity (three-component systems).

The number of possible binary systems is determined by the number of selected components. A total of 351 binary systems are formed from 27 “laser” metal fluorides (without Pm). Phase diagrams have been studied for more than 200 *M*F_m_–*R*F_n_ systems. This is sufficient to characterize the possibility of obtaining crystalline two-component materials in most chemical systems. A total of 480 new two-component fluoride phases are identified in the studied *M*F_m_–*R*F_n_ systems. Of these, 370 can be obtained in the form of single crystals.

Many two-component crystals exhibit large areas of homogeneity. These are required to control the operational properties of multicomponent fluoride crystals over a wide range. The most effective management method is controlling violations of the structure by strong nonstoichiometry. This is caused by the aliovalent isomorphism in *M*F_m_–*R*F_n_ (m ≠ n) systems. The *Spes-zd Emp* SIR is required for the creation and refinement of the structural models of the nonstoichiometric crystals for the prediction of their properties.

Among the cations with different valences (m ≠ n), approximately half do not belong to *Ln*. *M*F, *M*F_2_ and *M*F_4_ fluorides with *M* = Li, Na, K, Rb, Ca, Sr, Ba, Cd, Pb, Zr, Hf, and Th can be added to the studied 16 (*R*,*Ln*)F_3_ to obtain nonstoichiometric phases. The creation of an extended *Sp-zd Emp* SIR for phases in *M*F_m_–*R*F_n_ systems (m ≤ n ≤ 4) is the subject of consideration in the third part of this series of publications. It will present an extension of the *Sp-zd Emp* SIR for (La,*Ln*)F_3_ to additional *M*^n+^ cations (n = 1, 2, 4), which are not subject to LC and can be part of nonstoichiometric crystals. The calculation of the extended version of the SIR is supposed to be carried out in a similar empirical way based on the shortest (*M*-F)_min_ distances using the base radius of the fluorine anion ***r***_−_ = 1.2539(16) Å defined in this study.

## 3. Methods and Materials

### 3.1. Obtaining t-RF_3_ (R = La–Nd), “pseudo t-SmF_3_”, and β-LnF_3_ (Ln = Sm-Dy) for Structural Studies

To study the phase diagrams and structures of their components, ***t***-*R*F_3_ (*R* = La-Nd) and *β*-*Ln*F_3_ (*Ln* = Sm, Gd-Dy) reagents were prepared at the experimental plant (in the town of Pyshma) of the Government Institute of Rare Metals (GIREDMET, Moscow, Russia). (*R*,*Ln*)F_3_ were melted and fluorinated to purify oxygen impurities. Oxygen content of 0.005–0.08 wt.% was achieved (determination by vacuum melting [30,31,32]. After fluorination, the samples consisted of large crystalline blocks suitable for structural analysis.

A *β*-EuF_3_ reagent of high purity for radioactive impurities was used. It was obtained by the National Research Nuclear University “MEPhI” under the program to search for the double beta decay of _48_Ca in CaF_2_:Eu scintillator crystals.

The stabilization of the structural type ***t***- in SmF_3_ was carried out by adding 0.5 mol.% LaF_3_ [33]. The composition of Sm_0.995_La_0.005_F_3_ was further designated “*pseudo* ***t***-SmF_3_”.

### 3.2. X-ray Diffraction Study of t-RF_3_ (R = La–Nd), “pseudo t-SmF_3_”, and β-LnF_3_ (Ln = Sm-Dy) Crystals

A single crystal X-ray diffraction (**XRD**) study of ***t***-*R*F_3_ (*R* = La-Nd), “*pseudo* ***t***-SmF_3_”, and ***β***-*Ln*F_3_ (*Ln* = Sm-Dy) was performed at 293 K using an XtaLAB Synergy-DW (Rigaku Oxford Diffraction, Tokyo, Japan-Oxford, UK-Wroclaw, Poland) diffractometer with an Ag-anode X-ray tube. The data were processed using the CrysAlisPro version 171.42.72 (Rigaku Oxford Diffraction, Tokyo, Japan-Oxford, UK-Wroclaw, Poland) software package.

Powder XRD analysis was performed at 293 K with a Rigaku MiniFlex 600 Bragg-Brentano diffractometer using a Cu-anode X-ray tube within the range of 2θ = 10–100° and with a 2θ step size of 0.02°. A NIST 640e standard (Si) sample was added to the samples to determine the 2θ correction.

The details of the XRD experiments are listed in Table 3 and Table 4.

The JANA2020 program [34] was used for the structure solution and refinement. The structures were refined within *P-3c*_1_ (for the ***t***-type) and *Pnma* (for the ***β***-type) sp. grs. An isotropic extinction correction was introduced into the fitted models according to the Becker–Coppens formalism [35]. The Wyckoff positions (W.p.) [36], coordinates, site occupancy factors, and equivalent atomic displacement parameters for ***t***-*R*F_3_ (*R* = La-Nd), “*pseudo* ***t****-*SmF_3_”, and ***β***-*Ln*F_3_ (*Ln* = Sm-Dy) are listed in Table 5.

**Figure 6:**
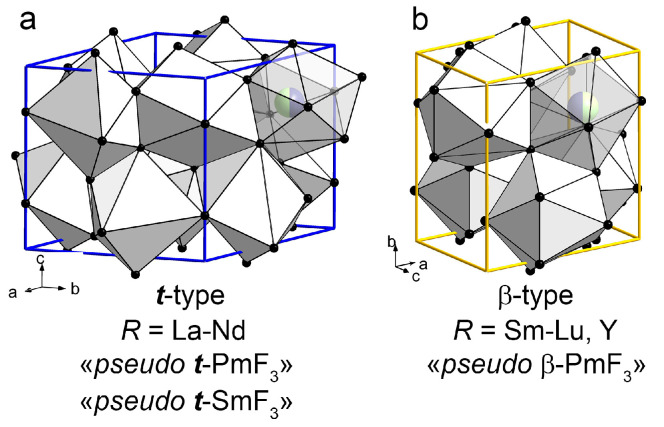
The structures of (**a**) ***t****-R*F_3_ (*R* = La-Nd), “*pseudo* ***t***-PmF_3_”, “*pseudo* ***t***-SmF_3_”, (**b**) “*pseudo* ***β***-PmF_3_”, and ***β****-R*F_3_ (*R* = Sm-Lu, Y). The unit cells of the ***t***- and ***β***-type structures are shown in blue and yellow respectively.

The ***t***- and ***β***-type structures are shown in Figure 6. *R*F_3_ crystals with *R* = La-Nd have a ***t***-type structure (Figure 6a). PmF_3_ and SmF_3_ are dimorphic. The PmF_3_ model, “*pseudo* PmF_3_”, crystalize in both ***t***- and ***β***-structural types (Figure 6a,b). The model of ***t***-SmF_3_, “*pseudo* ***t***-SmF_3_” has a ***t***-type structure (Figure 6a) and SmF_3_ can be obtained from melt in the ***β***-form (Figure 6b). *R*F_3_ crystals with *R* = Sm-Nd have a ***β***-type structure (Figure 6b).

## 4. Conclusions

The *Sp-zd Emp* SIR of Y^3+^, La^3+^, *Ln*^3+^, and F^1−^ for *R*F_3_ is derived from the precise LC dependence on Z in *Ln*F_3_. This is based on the empirical shortest (F-F)_min_ and (*R*-F)_min_. These distances are obtained from the structural data of the ***t***-*R*F_3_ (*R* = La-Nd), “*pseudo* ***t***-PmF_3_”, “*pseudo* ***t***-SmF_3_”, “*pseudo* ***β***-PmF_3_”, ***β***-*Ln*F_3_ (*Ln* = Sm-Lu), and YF_3_ crystals synthesized under the same conditions.

The accuracy of studying LC is determined by standard deviation (±0.0017 Å) of determining (*R*-F)_min_ and (F-F)_min_ in *R*F_3_ crystals by structural analysis. This accuracy is an order of magnitude higher than that claimed for *Univ* SIRs ±(0.01–0.02) Å.

The radius of the fluorine anion, ***r***_−_ = 1.2539(16) Å, is defined as half the arithmetic mean of five (F-F)_min_ for *Ln*F_3_ with *Ln* from Ho to Lu. It is accepted as the basis for calculating the *Spes-zd Emp* SIR. The radii of Y^3+^, La^3+^, *Ln*^3+^, and F^1−^ in the *Spes-zd Emp* SIR are constants of each individual *R*F_3_. The *R*^3+^ radii do not depend on the structural type of *R*F_3_ and do not require corrections for CN.

The (*R*-F)_min_ dependence on Z is described by a third-degree polynomial:

(*R*-F)_min_ = −1.67322·10^−4^·Z^3^ + 0.03163·Z^2^ − 1.99992·Z + 44.6499.

The unsuitability of LC studies on large arrays of homologous series of *Ln* compounds with different chemical bonds is shown. The precise LC study in one *Ln*F_3_ homologous series has established periodic deviations of the dependence of (*Ln*-F)_min_ on Z from the linear law. This dependence details the LC mechanism.

LC reduces ***r***_+_ by 24% (percentage relative to less) from 1.1671(16) (La^3+^) to 0.9439(17) Å in Lu^3+^.

The areas of use of the *Spes-zd Emp* SIR for *R*F_3_ for materials based on REE fluorides are determined. The *Spes-zd* Emp SIR for *R*F_3_ can be extended for the application to a chemical class of ionic inorganic fluorides.

## Figures and Tables

**Table 1:** (F-F)_min_ and (*R*-F)_min_ in ***t***-*R*F_3_ (*R* = La-Nd), “*pseudo **t***-PmF_3_”, “*pseudo **t**-*SmF_3_”, “*pseudo **β***-PmF_3_”, ***β***-YF_3_, and ***β***-*Ln*F_3_ (*Ln* = Sm-Lu) (this work and [1,10]).

*R*F_3_	(F-F)_min_, Å	(*R*-F)_min_, Å
***t***-LaF_3_	2.5615(14)	2.4201(4)
***t***-CeF_3_	2.5584(16)	2.3976(4)
***t***-PrF_3_	2.5496(9)	2.3787(3)
***t****-*NdF_3_	2.5340(8)	2.3613(3)
“*pseudo **t***-PmF_3_”	2.5363(15)	2.3542(3)
“*pseudo **β***-PmF_3_”	2.557(4)	2.349(4)
“*pseudo **t***-SmF_3_”	2.5154(18)	2.3432(4)
***β***-SmF_3_	2.5575(17)	2.3478(12)
***β***-EuF_3_	2.559(2)	2.3437(17)
***β***-GdF_3_	2.5474(14)	2.3353(10)
***β****-*TbF_3_	2.5398(16)	2.3234(10)
***β***-DyF_3_	2.5161(14)	2.3113(17)
***β***-HoF_3_	2.5064(15)	2.2986(14)
***β***-ErF_3_	2.5072(12)	2.2809(13)
***β***-TmF_3_	2.5079(14)	2.2513(15)
***β***-YbF_3_	2.5086(15)	2.2290(12)
***β***-LuF_3_	2.5089(16)	2.1969(17)
***β***-YF_3_	2.5130(9)	2.2877(11)

**Table 2:** The radii ***r***_+_ of *R*^3+^ cations (*R* = Y, La, and 14 *Ln*) in *R*F_3_.

*R* ^3+^	Struct. Type	*r*_+_, Å	*R* ^3+^	Struct. Type	*r*_+_, Å
La	***t*** *-*	1.1671(16)	Gd	***β***-	1.0823(16)
Ce		1.1446(16)	Tb		1.0704(16)
Pr		1.1257(16)	Dy		1.0583(17)
Nd		1.1083(16)	Ho		1.0456(16)
“*pseudo **t***-Pm”		1.1012(16)	Er		1.0279(16)
“*pseudo **β***-Pm”	***β***-	1.096(4)	Tm		0.9983(16)
“*pseudo **t***-Sm”	***t*** *-*	1.0902(18)	Yb		0.9760(16)
Sm	***β***-	1.0948(17)	Lu		0.9439(17)
Eu		1.0907(17)	Y		1.0347(16)

**Table 3:** Crystallographic characteristics and results of a structure refinement for ***t-****R*F_3_ (*R* = LaF_3_-NdF_3_) and “*pseudo **t***-SmF_3_” at 293 K.

*R*F_3_	*t*-LaF_3_	*t*-CeF_3_	*t*-PrF_3_	*t*-NdF_3_	“*pseudo* *t*-SmF_3_”
ICSD ID	2253935	2253989	2253990	2254005	2267804
Crystal system	Trigonal				
Sp.gr., Z	*P-3c*_1_, z = 6				
*a* (Å)	7.1859(2)	7.1296(3)	7.0780(2)	7.0298(3)	6.9626(7)
*c* (Å)	7.3543(2)	7.2867(4)	7.2392(3)	7.1988(3)	7.1317(8)
*V* (Å ^3^)	328.88(2)	320.77(3)	314.08(2)	308.09(3)	299.41(7)
V_form_	54.812	53.462	52.347	51.348	49.901
*D_x_* (g·cm^−3^)	5.9347	6.1225	6.2778	6.5077	6.8980
μ (mm^−1^)	10.167	11.154	12.194	13.288	15.535
*T_min_*, *T_max_*	0.2007, 0.2909	0.1806, 0.2707	0.1824, 0.2723	0.1345, 0.2306	0.0856, 0.1845
Shape, color	colorless	colorless	light green	lilac	light yellow
Diameter (mm)	0.22	0.22	0.20	0.22	0.115
Wavelength (Å)	0.56087				
Θ range (deg)	2.58–75.85	2.6–72.77	2.62–72.85	2.64–72.8	2.67–72.83
Refl. collected	34,709	33,176	32,968	32,720	31,459
Refl. unique/*R*_int_	4760/4.11	4438/4.20	4358/4.21	4267/4.76	3947
Refin. method	Full matrix least squares on F				
Param/Restrains	22/0	22/0	22/0	22/0	35/0
*R*/*wR*, %	2.06/3.60	1.71/4.03	1.79/3.25	1.73/2.85	2.01/3.15
Δρ_min_/Δρ_max_, Å^−3^	−2.07/2.39	−2.30/1.19	−3.28/2.16	−1.58/2.23	−2.71/1.17
GOF	1.22	1.51	1.14	1.03	0.97
Twins’ fractions	0.645(3)/ 0.355(3)	0.929(3)/ 0.071(3)	0.0398(18)/ 0.9602(18)	0.0190(17)/ 0.9810(17)	-

**Table 4:** Crystallographic characteristics and results of a structure refinement of ***β***-*Ln*F_3_ (*Ln* = Sm-Dy) at 293 K.

*Ln*F_3_	*β*-SmF_3_	*β*-EuF_3_	*β*-GdF_3_	*β*-TbF_3_	*β*-DyF_3_
ICSD ID	2254295	2254410	2254301	2254298	2254299
Crystal system	orthorhombic				
Sp.gr., Z	*Pnma*, 4				
*a* (Å)	6.6964(8)	6.6228(4)	6.5733(4)	6.5093(3)	6.4561(3)
*b* (Å)	7.0713(5)	7.0181(5)	6.9856(4)	6.9458(3)	6.9066(4)
*c* (Å)	4.3855(9)	4.3956(3)	4.3898(2)	4.3875(2)	4.3797(2)
*V* (Å ^3^)	207.66(5)	204.31(2)	201.573(19)	198.369(15)	195.290(17)
V_form_	51.916	51.076	50.393	49.592	48.822
*D_x_* (g·cm^−3^)	6.6323	6.7933	7.0597	7.2298	7.4654
μ (mm^−1^)	14.953	16.128	17.469	18.860	20.345
*T_min_*, *T_max_*	0.724, 1.469 empirical	0.614, 1.646 empirical	0.1173, 0.215	0.0816, 0.1804	0.0816, 0.1804
Shape, color	light yellow	light rose	colorless	colorless	colorless
Sample size, max/mid/min (mm)	0.191/ 0.133/ 0.042	0.268/ 0.163/ 0.121	0.18, diameter	0.20, diameter	0.20, diameter
Wavelength (Å)	0.56087				
Θ range (deg)	4.32–75.14	4.32–74.71	4.33–72.83	4.34–72.79	4.35–72.82
Refl. collected	172,260	163,839	21,098	21,136	20,682
Refl. unique/*R*_int_	4527/4.50	4472/6.05	3876/4.52	3595/4.96	3700/4.96
Refin. method	Full matrix least squares on F				
Param/Restrains	23/0	38/0	23/0	23/0	23/0
*R*/*wR*, %	2.11/3.92	3.28/4.95	2.32/3.74	2.36/3.57	2.45/3.78
Δρ_min_/Δρ_max_, Å^−3^	−2.60/1.98	−3.62/4.17	−3.59/4.02	−2.57/4.39	−4.17/4.68
GOF	0.85	1.04	1.49	1.48	1.46

**Table 5:** Wyckoff positions (W.p.), site occupancy factors (s.o.f.), fractional coordinates, and equivalent thermal displacement parameters of atoms in ***t*-***R*F_3_ (*R* = La-Nd), “*pseudo **t**-*SmF_3_”, and ***β-****Ln*F_3_ (*R* = Sm-Dy) at 293 K.

*R*F_3_	Ion	W.p.	s.o.f.	*x*/*a*	*y*/*b*	*z*/*c*	U_eq_
***t****-*LaF_3_	La	6*f*	1	0.339998(8)	0	1/4	0.006504(11)
	F(1)	12*g*	1	0.3653(2)	0.0526(2)	0.58164(13)	0.0160(4)
	F(2)	4*d*	1	1/3	2/3	0.1845(3)	0.0128(2)
	F(3)	2*a*	1	0	0	1/4	0.0261(8)
***t****-*CeF_3_	Ce	6*f*	1	0.340509(9)	0	1/4	0.006236(12)
	F(1)	12*g*	1	0.3672(3)	0.0552(2)	0.58078(15)	0.0150(3)
	F(2)	4*d*	1	1/3	2/3	0.1857(3)	0.0115(2)
	F(3)	2*a*	1	0	0	1/4	0.0222(7)
***t****-*PrF_3_	Pr	6*f*	1	0.341071(8)	0	1/4	0.006086(10)
	F(1)	12*g*	1	0.3678(2)	0.05667(18)	0.58057(8)	0.0141(2)
	F(2)	4*d*	1	1/3	2/3	0.1855(2)	0.01157(15)
	F(3)	2*a*	1	0	0	1/4	0.0208(6)
***t****-*NdF_3_	Nd	6*f*	1	0.341508(7)	0	1/4	0.006144(10)
	F(1)	12*g*	1	0.36866(16)	0.05757(14)	0.58086(8)	0.0134(2)
	F(2)	4*d*	1	1/3	2/3	0.18539(18)	0.01138(14)
	F(3)	2*a*	1	0	0	1/4	0.0200(5)
“*pseudo* ***t****-*SmF_3_”	Sm	6*f*	0.995	0.34030(2)	0	1/4	0.00772(5)
	La	6*f*	0.005				
	F(1)	12*g*	1	0.3691(3)	0.0580(3)	0.58021(16)	0.0187(4)
	F(2)	4*d*	1	1/3	2/3	0.1846(3)	0.0137(2)
	F(3)	2*a*	1	0	0	1/4	0.0285(8)
***β***-SmF_3_	Sm	4*c*	1	0.366109(13)	1/4	0.062105(18)	0.005419(10)
	F(1)	4*c*	1	0.5195(3)	1/4	0.5778(4)	0.0109(2)
	F(2)	8*b*	1	0.16446(18)	0.06374(17)	0.3932(3)	0.01010(14)
***β***-EuF_3_	Eu	4*c*	1	0.36668(3)	1/4	0.06272(6)	0.00649(5)
	F(1)	4*c*	1	0.5199(4)	1/4	0.5802(6)	0.0127(4)
	F(2)	8*b*	1	0.1652(2)	0.0645(2)	0.3897(4)	0.0108(2)
***β***-GdF_3_	Gd	4*c*	1	0.367519(9)	1/4	0.063353(13)	0.005183(10)
	F(1)	4*c*	1	0.5190(2)	1/4	0.5806(3)	0.00944(15)
	F(2)	8*b*	1	0.16517(14)	0.06404(14)	0.3878(3)	0.00937(10)
***β***-TbF_3_	Tb	4*c*	1	0.367331(9)	1/4	0.061873(13)	0.005439(10)
	F(1)	4*c*	1	0.5213(3)	1/4	0.5841(3)	0.01023(16)
	F(2)	8*b*	1	0.16517(18)	0.06421(12)	0.3842(2)	0.00948(10)
***β***-DyF_3_	Dy	4*c*	1	0.367890(9)	1/4	0.062002(16)	0.005198(11)
	F(1)	4*c*	1	0.5220(3)	1/4	0.5857(4)	0.0104(2)
	F(2)	8*b*	1	0.16413(14)	0.06354(14)	0.3819(3)	0.00888(11)

## Data Availability

The data presented in this study are available on request from the corresponding author. The data on the crystal structures are deposited in the Cambridge Structural Database (CSD num. 2253935, 2253989, 2253990, 2254005, 2267804, 2254295, 2254410, 2254301, 2254298, 2254299).
